# From Lark to Owl: developmental changes in morningness-eveningness from new-borns to early adulthood

**DOI:** 10.1038/srep45874

**Published:** 2017-04-05

**Authors:** Christoph Randler, Corina Faßl, Nadine Kalb

**Affiliations:** 1 University of Tuebingen, Department of Biology, Auf der Morgenstelle 24, D-70726 Tuebingen, Germany

## Abstract

Morningness-eveningness or chronotype changes significantly throughout the life span. This has been reported for the transition during adolescence in some studies, and to a lesser extent in early adulthood. Primary and pre-school children have been under investigation in fewer studies. This is the first comprehensive study covering the age range from very young children until early adulthood (0–30 years) based on the same measurement instrument. Here, we show that the turn towards eveningness starts at an early age in German children. Based on 26,214 cross-sectional data, we further show that at the end of adolescence, morningness-eveningness does not significantly change during early adulthood. Sex differences arise during puberty and remain until 30 years. The breaking point for the turn towards morningness is 15.7 years in girls and 17.2 boys. At the age of 0–1 years, there are about 70% morning types, and about 1% evening types, while at the age of 16 years, only 5% are morning types and 19% are evening types.

Morningness-eveningness or chronotype is an individual difference trait^1^. This trait refers to the sleep-wake behaviour (preferred bed times and wake times), as well as to times preferred for peak cognitive and physical performance and to psychological aspects, such as affect (e.g., the feeling after awakening). Some people are early risers that get up early, but go to bed early in contrast to people that get up late and go to bed late. Morning types (sometimes colloquially labelled as ‘larks’) usually feel refreshed soon after awakening and have their peak cognitive performance in the morning. Evening types (colloquially named as ‘owls’) need more time to feel refreshed after awakening and they have their peak performance in the late afternoon or at night (see Adan *et al*. for an overview^1^). There are different operationalisations of morningness-eveningness and chronotype, although they are moderately correlated^1^. Morningness-eveningness refers to a preference for given times for bed and waking times, while chronotype is mainly based on self-report clock times for waking and bed times on free and scheduled days^2^. The morningness-eveningness scales measure a broader construct, including variables such as morning affect and peak performance^1^. In this study, we focus on the morningness-eveningness scales (see below), because they are the most widely used questionnaires worldwide to assess this trait.

Morningness-eveningness affects many different aspects in our daily life and it is determined by a variety of factors. There are biological factors (specific genes^3^), individual factors (e.g., age, sex^1^) and environmental factors (e.g., changes of light and dark, latitudinal influence^4^). In addition, social factors also influence chronotype (e.g., time schedules in school, shift work, lifestyle^5^).

This basic biological trait is usually assessed by questionnaires in survey studies^6^. The scores on the questionnaires are correlated with dim light melatonin onset^7^, cortisol levels in the morning^8^, as well as with circadian genes^1^. Moreover, the scores on the questionnaires are correlated with objectively measured sleep-wake behaviour^9^. Thus, we can use these questionnaires as reliable and valid instruments to assess morningness-eveningness in large-scale survey studies.

Morningness-eveningness changes significantly during the lifespan^10^. This has been shown in a few studies based on reasonable datasets. For example, Roenneberg *et al*.^2^ showed that midpoint of sleep on free days becomes increasingly later from 10 years of age until about 20 years when the peak of lateness is reached. Then people become earlier chronotypes again until older age. Although based on an impressive database (N = 25,000), children below 10 years are not shown in detail. Other large-scale studies also focused mainly on adolescents and adults (Finland: Merikanto *et al*.: N = 6,858^11^; age range 25–74; Brazil: Duarte *et al*.: N = 14,650; from ≈20 to >60 years^12^; Italy: Tonetti *et al*.: N = 8,972, 10–87 years^13^; Germany: Randler *et al*., N = 14,987; 5–70 years^14^).

In adolescents, Borisenkov *et al*.^15^ showed a significant phase delay between 11 and 22 years (N = 1101; Russia). Russo *et al*.^16^ (based on 1073 Italian adolescents aged 8–14 years) reported an increasing eveningness with the most obvious difference between age 12 and 13. Collado *et al*. (N = 2.649^17^) showed that Spanish adolescents between 12 and 16 years increasingly become evening types. Beal *et al*.^18^, based on a longitudinal study with 262 American girls aged 11–19 years, showed that girls are more evening oriented as they approached menarche and afterwards, no further change to eveningness occurred.

Studies focusing on young children are usually based on smaller samples and/or restricted age ranges. For example, Doi *et al*.^19^ based their research on an impressive dataset of 7826 Japanese pre-schoolers (age 3–5 years). These authors found no influence of age on children’s chronotype. Similar results have been reported by Werner *et al*. (N = 152^20^) for Switzerland. Zimmermann (N = 529; USA^21^), however, showed that already a shift in younger age towards eveningness is visible (e.g., between the ages of 2 and 4 years; see also Randler and Truc (2014; N = 199, for Germany^22^). Based on this literature review, studies are lacking that link early childhood with adolescence and early adulthood.

Sex differences in children and adolescents have been addressed in some studies. In young children, some studies^19^,^21^,^23^ found no influence of sex, while others reported no data on this, suggesting there may be no differences between the sexes^24^,^25^,^26^. Concerning adolescents, some studies reported a higher morning orientation in girls^27^, and others a higher morningness in boys around the age 13–14 years^17^,^28^. Also, some studies revealed no differences^16^,^29^. These inconsistent results request further studies. The differences between studies may be owed to two factors: i) the sample size may have been too low to detect small differences, or ii) the interaction effects between age and sex may have a masking effect on the general picture (see Duarte *et al*.^12^ for adults). Thus, studies should provide a high sample size to investigate this aspect.

Different approaches can be used to assess morningness-eveningness. First, we used the full score of the Composite Scale of Morningness (CSM) with 13 questions as well as a categorical classification into morning, evening and neither types. This classification has been established because it separates people into the different groups following cut-off scores. Second, we focus on the single item (self-assessment) as suggested by Loureiro and Garcia-Marques^30^. Some researchers emphasize that even this single item is already sufficient for a thorough analysis^30^, because people seem to be able to make a reliable self-assessment of their own chronotype^31^.

This Study Has Three Aims. First, it fills the gap between young age and early adulthood (about 30 years) in addressing morningness-eveningness changes in a cross-sectional manner across a wide age range. Second, the study provides a high sample size to assess differences between boys and girls for every age group separately. Third, different measurements were used to demonstrate the age-related changes in morningness-eveningness, such as the total CSM scores, a group classification, and the single item measurement.

## Results

**Figure 1:**
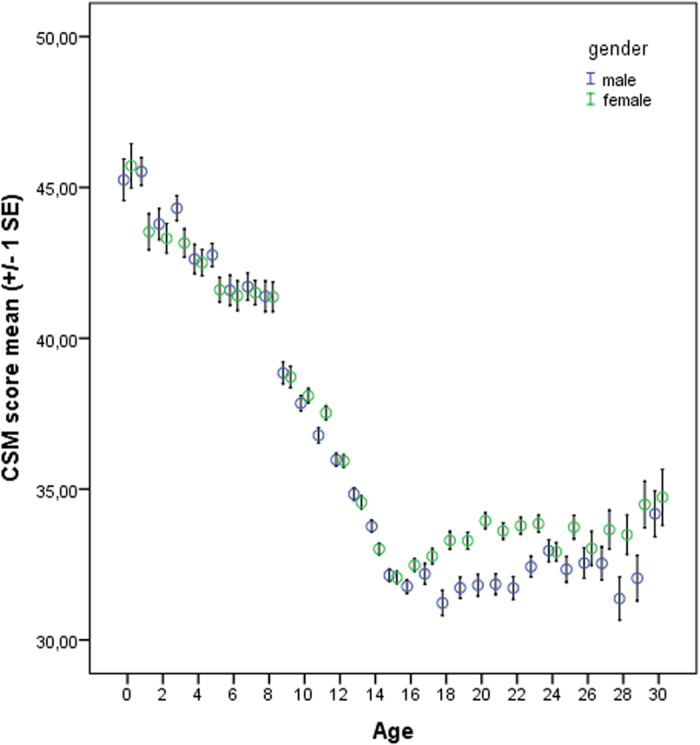
Distribution of morningness-eveningness across age groups based on the Composite Scale of Morningness total scores. Higher scores indicate higher morningness.

**Figure 2:**
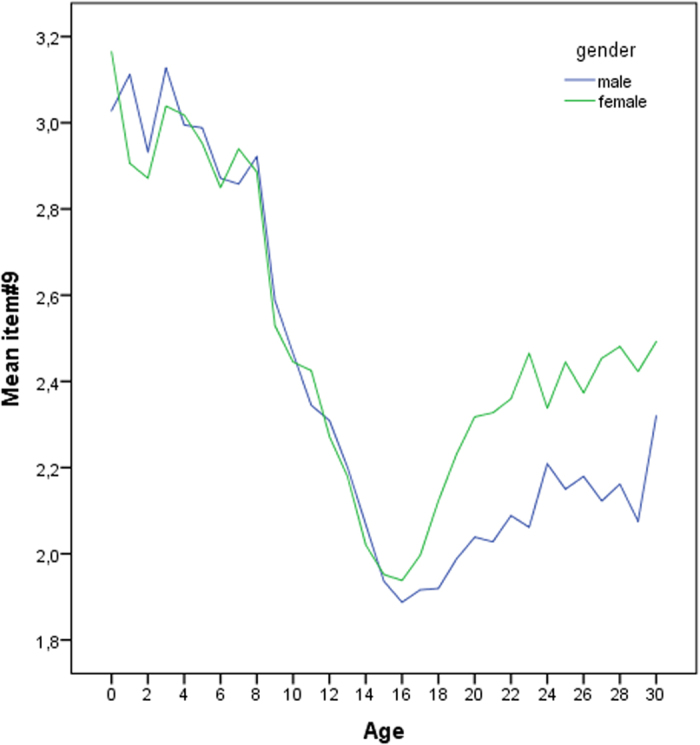
Distribution of morningness-eveningness across age groups based on the self-assessment item (item 9; scores ranging from 1–4). Higher scores indicate higher morningness.

**Figure 3:**
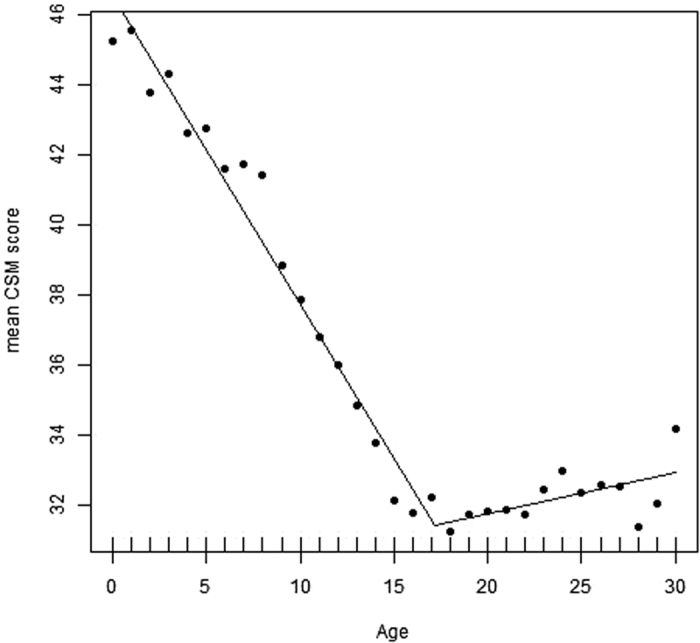
Segmented regression showing the breaking point for boys. Higher scores indicate higher morningness.

**Figure 4:**
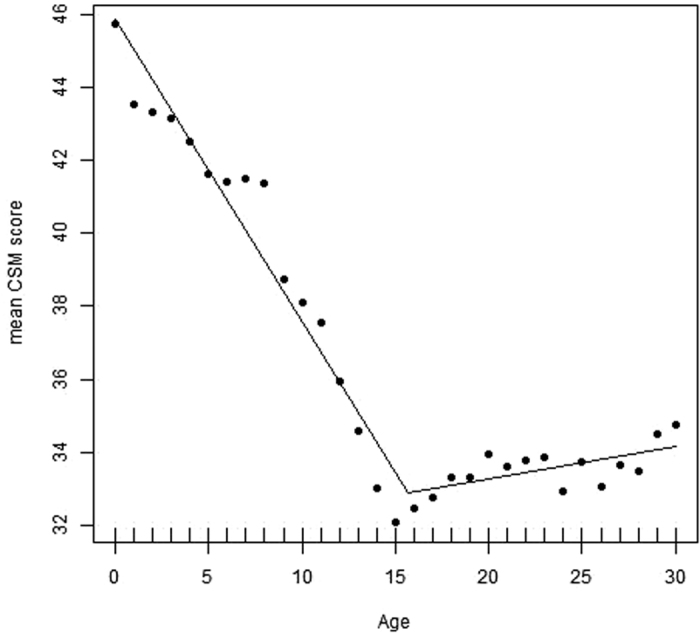
Segmented regression showing the breaking point for girls. Higher scores indicate higher morningness.

Mean CSM scores (mean ± SD) were 35.16 ± 7.46 which is near the scale mean of 34. Figure 1 shows the relationship between age and morningness-eveningness from 0–30 years based on the CSM scores, and the self-assessment item according to sex/gender (Fig. 2). Morningness already decreases right from the beginning. Age differences were significant (p < 0.05) between the age groups 1–2, 3–4, 8–9, 9–10, 10–11, 11–12, 12–13, 13–14, 14–15, and then remained more or less on the same level. Thus, a slight turn towards eveningness occurs already during toddler age. However, the greatest change occurs during pre-puberty around the age of 9–10 years. From 16–17 years, morningness-eveningness stabilizes on a low level (eveningness). This is reflected both, in the CSM scores as well as in the self-assessment item. Boys reach their nadir and their peak of lateness at the age of 18 years, girls at the age of 15 years. Based on the self-assessment item (item #9) the nadir is 16 years for both sexes. Based on the segmented regression, the breaking point in boys is at 17.2 ± 0.57 years (Fig. 3; Intercept: 46.531; x = −0.879; difference in slope parameter for the variable age = 0.995; multiple R-squared: 0.978, adjusted R-squared: 0.976) and in girls at 15.65 ± years (Fig. 4; Intercept 45.841; x = −0.828; difference in slope parameter for the variable age = 0.918; multiple R-squared: 0.967, adjusted R-squared: 0.964). Table 1 shows the age-related differences according to the classification into morning types, neither types and evening types. While evening types are nearly absent during young age, morning types progressively become rarer and the percentage of evening types increases.

Sex differences were assessed for every age group to detect interactions on CSM scores. Sex differences existed in the following age groups: Boys were higher on morningness at the age of 1 year, of 5 years and of 14 years (p < 0.05 for all comparisons). Girls scored higher on morningness at the age of 11 years, 16 years, 18 years and 19 years. In early adulthood, women were higher on morningness at the age of 20, 21, 22, 23, 25, 28, and 29 (p < 0.05 for all comparisons). Thus, in early adulthood clear differences between the sexes exist, while there are interaction effects during childhood. Concerning the self-assessment item, differences between men and women existed from 18–29 years, with women always scoring higher (more morning oriented; p ≤ 0.05). Requests for individual findings can be obtained by contacting the corresponding author.

## Discussion

Four main findings arise from this study. First, the transition towards eveningness already starts in early childhood. Second, the peak of eveningness is at around the age of 16 years in girls and of 17 years in boys. Third, after this peak in eveningness, morningness-eveningness shows no differences between the subsequent age groups. Fourth, sex differences occur especially around the age 16 onwards and remain during early adulthood. These findings will be discussed in turn.

Morningness already decreases right from the beginning (age 1–2 and 3–4), a finding that has been found by Zimmermann^21^. Similarly, Nakade^24^ reported that 2-year-olds had higher morningness scores compared to 3 to 5-year-olds. Wada^25^ mentioned that infants in the Czech Republic and in Japan became more evening oriented according to age. Wickersham^26^ reported that 2- and 3-year-olds show extreme morning tendencies, with roughly 90% of all children scoring as morning types. This is different from our 60–65% in this age group, but may be a result of different assessment methods. Another aspect might be that Wickersham^26^ did the study in the USA or may lie in different cut-off criteria.

In addition to previous work, our sample allows us to detect differences between newborns, children, adolescents and early adulthood in one single study. This is a new and important finding. While most other studies give snapshots of the development, we here cover the full spectrum before and after puberty/adolescence until early adulthood. Similarly, we detected a strong turn to eveningness already at the age of 9 years, a result that has gone unnoticed in previous work (also mostly due to the smaller age ranges in these studies). This turn towards eveningness at around 9 years may also result from the parent versus the self-report data. For younger kids, the parents have estimated the chronotype until the age of 9/10 years while self-report was used from age 10/11. We based the separation parent- versus self-report on grade 4, thus from grade 4 onwards, participants self-reported their data. Unfortunately, we are not able to find any study that compared self-and parent report together with each other (this has been done in some psychological studies on personality traits etc., but we are not aware of such a study in morningness-eveningness). However, the general pattern seems not to be influenced by these measurements because the general pattern is clear, namely a decrease in morningness. Therefore, only the steepness might be under discussion. Future studies should include a comparison of parent- versus self-report in adolescents.

The peak of eveningness is roughly around the age of 16 years, which is in contrast to other German data^2^. Some studies showed that people even become later types until the age of 22 years^15^. However, three facts might contribute to the differences. First, these studies sampled different populations and cultures^15^, and culture may have a general influence on changes in the sleep-wake cycle. Second, we assessed a large age range to reflect the population changes from the turn into eveningness and back towards morningness; in fact, this is the first study covering such a large age range. Third, different measures of chronotype may affect the results, which especially may explain the differences between Roenneberg’s^2^ and Borisenkov’s^15^ work. These authors used a clock-based measure, the MCTQ, while Beal *et al*.^18^ and we used a preference measure.

Concerning girls and women, Frey *et al*.^32^ detected the nadir of lateness around 5 years after menarche (at about 13 years in their study) which represents an age of about 17–18 years for the peak of lateness^32^. This is somewhat later than in our study. However, Beal *et al*.^18^ showed that after menarche no change in morningness-eveningness occurs anymore. These results are contradictory and need further work to be clarified. However, our results show that the evening lateness is in between these two studies with an age of about 15.65 years. The results may differ because of two important aspects: The sample size in Beal *et al*.^18^ is somewhat small (N = 292, but it is a longitudinal study, which is very rare). Our data have been obtained based on a questionnaire comparable to Beal *et al*.^18^ while Frey *et al*.^32^ is based their study on a clock-time measurement. This might be the reason why our breaking point is closer to Beal *et al*.^18^ because there may be differences in assessment of clock-based chronotypes versus morningness-eveningness preference.

Based on the segmented regressions, the breaking point in boys was at 17.2 years and at 15.7 years in girls. The earlier breakpoint in girls might be related to their advanced maturation. Girls have an earlier onset of their pubertal development than boys^33^. Combined with the study findings of Beal *et al*.^18^ and Frey *et al*.^32^ we suppose that indeed puberty seems the trigger for a turn towards eveningness, especially in girls, where the relationship with menarche seems the most important factor^18^,^32^. However, this leads to the question why boys turn towards eveningness because they do not experience menarche. Here, the hormone testosterone may have a crucial role because in university students with an age range of 20–30 years, Randler *et al*.^34^ reported higher testosterone levels being associated with eveningness. As boys receive their sexual maturity, they turn back to morningness later than girls, and young men remain longer on eveningness, thus, the differences between men and women are largest during the reproductive period^2^,^14^. One possible cause might be that it is a sexually selected trait^35^ but evidence for that is still scarce. Our study further indicates that there are interaction effects between age and sex, which should be under investigation in the future to reveal which factors influence this interaction (biological, social, environmental).

Finally, the single item (item 9) measurement concerns a self-assessment of chronotype and our analyses based on this item (Fig. 1B) revealed the same result as the total scores of the CSM. This gives some evidence that single item measures could be a useful and informative tool when time is constraint. This was put forward by Turco *et al*.^36^, Loureira & Garcia-Marques^30^ and Simpkin *et al*.^23^. This is also corroborated by Furnham^31^ who asked if people are able to assess their own personality score. Furnham^31^ showed that subjects would best be able to predict factors like morningness-eveningness, extraversion and introversion.

## Methods

The measurement is based on the Composite Scale of Morningness, an instrument developed to assess morningness-eveningness^37^ (German versions: ref. 38). The scale has undergone some validation studies in adults and adolescents^6^,^39^. For example, the scores obtained on the scale are stable over a period of some months. Convergent validity was obtained with the Morningness-Eveningness-Questionnaire (MEQ; correlation of about. 9) and with its short form, the rMEQ (correlation of about. 8). Construct validity was obtained by actigraphy^9^, and the CSM scores correlated with about 0.5 with objectively measured bed and rise times^6^,^39^.

The CSM is a 13-item measurement with 10 items scaled from 1–4 and three items scaled from 1–5. The scores range from 13 to 55 with higher scores indicating higher morningness. The scores of the CSM can be used to calculate a raw score, but also to classify types. Individuals with a score of 26 and lower are classified as evening types (ET), and with a score of 43 and higher as morning types (MT). Individuals in between are labelled neither types (NT)^38^. Recent studies discussed the possibility of using only one single item for assessment of chronotype^30^,^36^. This refers to item 9 of the CSM, where people have to assess whether they are a morning or evening person. Although the quality of a single item measure is under discussion in psychological research, we applied this single item in addition to the analysis of the full scale. To get a complete picture, we applied the CSM with raw scores, and chronotype by using the cut-off scores and the self-assessment item (item 9 of the CSM).

The CSM can be used in all age groups. Self-report instruments have been used from grade 4 onwards^39^, while parents filled in questionnaires for younger children from grade 3 downwards^22^. The measurement is valid for use in kindergarten children, adolescents and adults^6^,^22^,^39^. Cronbach’s alpha in the current sample is 0.897 for small children, 0.886 for kindergarten children, 0.851 for school pupils, 0.874 for university students and 0.854 for adolescent/adult workers.

### Participants and data collection

The study is based on 26,214 participants (12,531 male, 13,683 female) between 0 and 30 years of age (mean = 14.68; SD = 6.04). Participants were recruited during the years from 2006 to 2016. Kosec *et al*.^40^ showed that morningness-eveningness scores remained stable over decades when comparing young university students, thus we assume no influence by this large sampling period. The data were collected by paper-pencil questionnaires (about 95%) and to a small extent by online questionnaires. The focus on paper-pencil-questionnaires was done to have some control over the population and the participants. We focused on the population in SW and S Germany and we did the survey in many different institutions, from pre-schoolers, kindergarten children, schools and universities. The sample is not representative, but the high number of participants renders this very likely, e.g., the schools covered all stratifications and school levels (according to the SW German school system, children usually are separated in grade 5 into 3–4 stratifications, like Werkrealschule, Realschule, Gymnasium and Förderschule). We covered all these school types. Given the recommendations of Krejcie & Morgan^41^, the sample size is high enough to cover a representative population. In total, more than 70 students participated in data collection for this study.

### Statistical analyses

SPSS 24.0 was used to perform T-tests to compare age groups and sex. We performed a segmented linear regression using the package segmented^42^,^43^ in R^44^ to analyse the relationship between age and mean CSM score in males and females. The package segmented is used to analyse linear models that show one or more segmented relationships in their linear predictor (in our case age) and provides the slopes and breakpoints of those relationships.

All protocols were approved by the University of Education Heidelberg following the guidelines of the Forschungskommission. The methods were carried out in accordance with the relevant guidelines and regulations. We have received informed consent of all participants as well as written informed consent of all participants’ parents when below 18 years.

## Additional Information

**How to cite this article:** Randler, C. *et al*. From Lark to Owl: developmental changes in morningness-eveningness from new-borns to early adulthood. *Sci. Rep.* **7**, 45874; doi: 10.1038/srep45874 (2017).

**Publisher's note:** Springer Nature remains neutral with regard to jurisdictional claims in published maps and institutional affiliations.

## Figures and Tables

**Table 1:** Overview over the samples according to age and classification of chronotype into morning, neither and evening types.

			Chronotype			Total
			ET	NT	MT	
Age	0.00	N	0	39	99	138
		%	0.0%	28.3%	71.7%	100.0%
	1.00	N	3	78	179	260
		%	1.2%	30.0%	68.8%	100.0%
	2.00	N	6	138	224	368
		%	1.6%	37.5%	60.9%	100.0%
	3.00	N	4	143	278	425
		%	0.9%	33.6%	65.4%	100.0%
	4.00	N	5	181	231	417
		%	1.2%	43.4%	55.4%	100.0%
	5.00	N	2	229	256	487
		%	0.4%	47.0%	52.6%	100.0%
	6.00	N	6	168	175	349
		%	1.7%	48.1%	50.1%	100.0%
	7.00	N	3	207	207	417
		%	0.7%	49.6%	49.6%	100.0%
	8.00	N	4	164	151	319
		%	1.3%	51.4%	47.3%	100.0%
	9.00	N	23	452	197	672
		%	3.4%	67.3%	29.3%	100.0%
	10.00	N	71	1053	398	1522
		%	4.7%	69.2%	26.1%	100.0%
	11.00	N	120	1224	376	1720
		%	7.0%	71.2%	21.9%	100.0%
	12.00	N	209	1659	399	2267
		%	9.2%	73.2%	17.6%	100.0%
	13.00	N	280	1654	267	2201
		%	12.7%	75.1%	12.1%	100.0%
	14.00	N	374	1791	210	2375
		%	15.7%	75.4%	8.8%	100.0%
	15.00	N	468	1565	142	2175
		%	21.5%	72.0%	6.5%	100.0%
	16.00	N	324	1322	94	1740
		%	18.6%	76.0%	5.4%	100.0%
	17.00	N	170	742	62	974
		%	17.5%	76.2%	6.4%	100.0%
	18.00	N	149	555	47	751
		%	19.8%	73.9%	6.3%	100.0%
	19.00	N	129	620	36	785
		%	16.4%	79.0%	4.6%	100.0%
	20.00	N	129	699	59	887
		%	14.5%	78.8%	6.7%	100.0%
	21.00	N	157	686	53	896
		%	17.5%	76.6%	5.9%	100.0%
	22.00	N	131	629	52	812
		%	16.1%	77.5%	6.4%	100.0%
	23.00	N	142	650	59	851
		%	16.7%	76.4%	6.9%	100.0%
	24.00	N	119	598	43	760
		%	15.7%	78.7%	5.7%	100.0%
	25.00	N	90	406	34	530
		%	17.0%	76.6%	6.4%	100.0%
	26.00	N	76	259	26	361
		%	21.1%	71.7%	7.2%	100.0%
	27.00	N	53	206	24	283
	28.00	%	18.7%	72.8%	8.5%	100.0%
		N	39	147	18	204
		%	19.1%	72.1%	8.8%	100.0%
	29.00	N	25	95	13	133
		%	18.8%	71.4%	9.8%	100.0%
	30.00	N	16	101	18	135
		%	11.9%	74.8%	13.3%	100.0%
Total		N	3327	18460	4427	26214
		%	12.7%	70.4%	16.9%	100.0%

ET = Evening types, NT = Neither types, MT = Morning types.
